# Tragic Choices and the Virtue of Techno-Responsibility Gaps

**DOI:** 10.1007/s13347-022-00519-1

**Published:** 2022-03-30

**Authors:** John Danaher

**Affiliations:** School of Law, NUI Galway, Galway, Ireland

**Keywords:** Responsibility, Tragic choices, Moral dilemmas, Delegation, Illusions, Burden of choice, Responsibility gap, AI, Robotics

## Abstract

There is a concern that the widespread deployment of autonomous machines will open up a number of ‘responsibility gaps’ throughout society. Various articulations of such techno-responsibility gaps have been proposed over the years, along with several potential solutions. Most of these solutions focus on ‘plugging’ or ‘dissolving’ the gaps. This paper offers an alternative perspective. It argues that techno-responsibility gaps are, sometimes, to be welcomed and that one of the advantages of autonomous machines is that they enable us to embrace certain kinds of responsibility gap. The argument is based on the idea that human morality is often tragic. We frequently confront situations in which competing moral considerations pull in different directions and it is impossible to perfectly balance these considerations. This heightens the burden of responsibility associated with our choices. We cope with the tragedy of moral choice in different ways. Sometimes we delude ourselves into thinking the choices we make were not tragic (illusionism); sometimes we delegate the tragic choice to others (delegation); sometimes we make the choice ourselves and bear the psychological consequences (responsibilisation). Each of these strategies has its benefits and costs. One potential advantage of autonomous machines is that they enable a reduced cost form of delegation. However, we only gain the advantage of this reduced cost if we accept that some techno-responsibility gaps are virtuous.

## Introduction

The rise of autonomous machines (AI and robotics) has raised a number of important ethical and legal concerns. The big three are fairness/bias, transparency/explainability and accountability/responsibility. This paper focuses on the last of these, which is typically expressed as the concern that the widespread deployment of autonomous machines will open up a number of ‘responsibility gaps’ throughout society. In essence, the concern boils down to this: 1

***Techno-Responsibility Gap Concern:*** As machines grow in their autonomous power (i.e. their ability to do things independently of human control or direction), they are likely to be causally responsible for positive and negative outcomes in the world. However, due to their properties, these machines cannot, or will not, be morally or legally responsible for these outcomes. This gives rise to a potential responsibility gap: where once it may have been possible to attribute these outcomes to a responsible agent, it no longer will be.

Various articulations of such techno-responsibility gaps have been proposed over the years, along with several potential solutions. Most of these solutions focus on strategies for ‘plugging’ or ‘dissolving’ the gaps (e.g. Kraaijeveld 2020; Pagallo 2011; Tigard 2021). In this article, I defend an alternative perspective on techno-responsibility gaps. Instead of these gaps being seen as things that need to be plugged or dissolved, I will argue that some responsibility gaps should be welcomed.

The gist of my argument is this: human life is replete with morally tragic choices. We often confront situations in which competing moral considerations pull in different directions. Many times it is impossible to satisfy each consideration in a single decision. This heightens the burden of responsibility associated with human decision-making. We cope with the tragedy of moral choice in different ways. Sometimes we convince ourselves that the choices we make are not tragic; sometimes we delegate the tragic choice to others; sometimes we make the choice ourselves and bear the psychological consequences. I call these three strategies: *illusionism*, *delegation* and *responsibilisation*, respectively. Each strategy has its own share of benefits and costs and one of the primary goals of this paper is to articulate, in some detail, what these benefits and costs might be. I then argue that the increased use of autonomous machine-based decision-making systems changes the balance of costs and benefits associated with these different strategies. In particular, it allows for a *reduced cost* form of delegation, which removes some of the psychological and moral costs associated with this strategy, and thus gives us some reason to shift away from illusionism and responsibilisation. If we wish to avail of this reduced cost form of delegation, we must embrace some techno-responsibility gaps.

It is important that this argument is not overinterpreted. I am not claiming that machine-induced responsibility gaps are always and everywhere a good thing, nor am I claiming that delegation to machines is always virtuous. I am only claiming that *sometimes* this is the case and it is a mistake to suppose that plugging or dissolving a techno-responsibility gap is always a good thing. Furthermore, I am not claiming that autonomous machines *necessarily* allow us to outsource the burden of tragic choice with reduced moral costs. Some autonomous systems’ architectures heighten the costs associated with tragic choices. I will return to this problem later in the article and suggest that it creates its own ‘meta-tragedy’ when it comes to the design of autonomous machines.

The paper will proceed in four main parts. First, I will briefly review some of the literature on techno-responsibility gaps and situate my own argument relative to that literature. Second, I will explain the problem of tragic choices (in the sense used in this article) and why it is worth taking seriously. Third, I will outline three strategies for coping with the problem of tragic choices, highlighting their respective benefits and drawbacks. Fourth, I will argue that a potential upside of autonomous machines is how they enable us to favour delegation over responsibilisation. In making this argument, I will respond to four major criticisms of it.

## Attitudes Toward Techno-Responsibility Gaps

Let’s start by briefly reviewing some of the existing literature on techno-responsibility gaps. This will help to explain how the argument developed in this article builds upon and differs from the arguments put forward in the literature to date.

The original formulation of the techno-responsibility gap concern can be found in a paper by Andreas Matthias (2004). This paper looks specifically at the challenge of ascribing responsibility for the actions of ‘learning automata’. Matthias’s main insight was this: ordinarily, if something goes wrong with a machine, we trace responsibility for that error back to the designers or, in some cases, owners and controllers of that machine. This makes sense given that they control the operating parameters of the machine. But, Matthias argued, the advent of machines that are not specifically programmed to act in a particular way but can, instead, learn from their experiences and interactions with a physical environment, undermines this traditional approach to responsibility. With learning automata, it no longer seems so correct to engage in this tracing strategy. Matthias framed his argument as a dilemma: either we embrace the creation and use of such learning machines, and resign ourselves to the fact that they will do things for which no one can be held responsible, or we reject their creation and use and thus forego their conveniences. Since Matthias originally formulated the concern, many others have sought to expand it and refine it. For example, Robert Sparrow (2007) formulated a version of the challenge that applied specifically to the use of autonomous weapons in war, arguing that the use of non-responsible autonomous systems would undermine the principles of just war and thus should not be allowed. John Danaher (2016) formulated a version of the challenge that applied specifically to the desire to seek retribution.

As might be expected, from these initial articulations, discussions of the techno-responsibility gap concern have become quite complex. Philosophers have long noted that the concept of responsibility is multiply ambiguous (Hart 1968; Vincent 2011). Poel and Sand (2018), for instance, have argued that there are at least five distinct types of responsibility operative in ethical and policy discussions. Some of these types of responsibility are backward-looking, focusing on responsibility for past events; some of them are forward-looking, focusing on taking responsibility for future actions.

A recent report on the ethics of autonomous vehicles, from the European Commission (Bonnefon et al. 2020), used this framework to suggest that there are, as a result, five distinct techno-responsibility gaps that arise from the use of autonomous machines: three backward-looking and two forward-looking. Looking backward, if an autonomous machine causes some harm, this can give rise to an *accountability gap* (no one to give public account for the harm done), a *culpability gap* (no one to morally blame for the harm) and a *compensation gap* (no one to pay damages for the harm). Looking-forward, the creation of these machines gives rise to a possible *obligation gap* (a danger that no one has the duty to ensure that the machine avoids or minimises harm) and a *virtue gap* (no one tries to cultivate a disposition to take responsibility for the actions of machines). Using a similar framework, Santoni de Sio and Mecacci (2021) have suggested that there are four distinct responsibility gaps arising from the use of autonomous machines, three backward-looking (culpability, moral accountability and public accountability) and one forward-looking (taking active responsibility).

If that were not enough, Sven Nyholm (forthcoming) has added further complexity to the discussion by suggesting that we should also consider the distinction between positive responsibility (i.e. being praised or rewarded for doing good or avoiding harm) and negative responsibility (i.e. being blamed or punished for doing bad or increasing risk). He uses this to suggest that there are negative and positive techno-responsibility gaps in the backward-looking direction as well as in the forward-looking direction (see also Danaher and Nyholm, 2021 on this idea).

Various solutions to the different techno-responsibility gaps have been proposed over the years. Most solutions share the common belief that the true extent of machine autonomy can be overstated and that the alleged gaps can, contrary to what Matthias suggested, be plugged by *tracking* and *tracing* the actions of machines back to humans (Santoni de Sio and Mecacci 2021; Santoni de Sio and Hoven 2018). In a similar vein, Nyholm (2017) argues that we can use models of collaborative agency or principal-agent relationships to understand how humans can be responsible for the actions of autonomous machines. Just as humans can be held responsible for the actions of other humans that they supervise and direct, so too can they be held responsible for the actions of machines that they supervise and direct. This is true even if they do not control the proximate causes of the machine’s actions. This builds on the insight developed by others which suggests that we can use the model of human responsibility for trained animals as a basis for tracing machine responsibility back to humans (Schaerer et al. 2009).

Other people reject Matthias’s original formulation of the problem argue that there is no gap that needs to be plugged. Daniel Tigard (2021), for instance, has argued that much of the discussion around techno-responsibility gaps is conceptually confused. If machines cannot be fitting subjects of responsibility judgments (blame, shame, etc.), and there are no associated humans to whom responsibility can be traced, then there is no gap that needs to be plugged (because there is no fitting subject of responsibility). In other words, the demand for some agent to be held responsible in these cases is illegitimate and ill-founded. If, on the other hand, responsibility can be traced to humans, there is no gap either since they can be held responsible for what the machines do. Tigard’s scepticism tries to dissolve much of the current debate. More controversially, Tigard suggests that it may be appropriate to attribute some kinds of responsibility to some machines. Building on this idea, and drawing from on his work on group agency and responsibility, Christian List (2021) has recently argued that it may be plausible to treat some AI entities as akin to group agents and attribute a certain form of moral and legal responsibility to them, thereby eliminating any alleged techno-responsibility gap.

What is interesting about the extant literature is that, for all its complexity, most contributors to the techno-responsibility gap debate tend to agree on one thing: the creation of techno-responsibility gaps is a problem. The primary reason for this is that responsibility tends to be seen as a good thing. This is inherent in Matthias’s original formulation of the challenge as a dilemma involving the tradeoff between different values: responsibility for machine-caused harms versus the efficiency and convenience of machines. It is also present in Sparrow’s discussion of the value of responsibility in a just war: we would fail to satisfy an important condition of justice if we employed such machines in the battlefield. But what is it that is so valuable about responsibility? Why should we worry about techno-responsibility gaps?

In answering this question, it is important to distinguish between the backward and forward-looking dimensions of responsibility. There are several reasons for thinking that backward-looking responsibility is a good thing. For instance, you could argue that retribution is an intrinsic good and so it is inherently good to punish those that are responsible for harmful outcomes (Danaher 2016; Moore 1993). Alternatively, you could argue that it is good for people to be compensated for any harms that befall them and tracing responsibility to an appropriate agent that can pay this compensation is a positive thing (Santoni de Sio and Mecacci 2021; Bonnefon et al. 2020). That said, it is worth noting that there are many moral critics of backward-looking responsibility and its associated social practices (e.g. Boonin 2008; Caruso 2021; Zimmerman 2011). These critics argue that our responsibility practices are often inherently unjust and serve as a front for cruel and unfair treatment of others. I will revisit some of their criticisms later in this article.

The forward-looking value of responsibility is, perhaps, easier to understand and less controversial. In our society, it is commonly assumed that it is a good thing to live up to your moral obligations and to take responsibility for your choices (e.g. Ruyter 2002; Williams 2008; Vincent 2011). The virtuous person is one that takes their responsibilities seriously and tries to be accountable to others (Torrance 2021). Even those that are otherwise critical of backward-looking responsibility tend to accept that there is some virtue in forward-looking responsibility (Caruso 2021). That said, the two forms of responsibility are linked and worries about forward-looking responsibility often spill over into worries about backward-looking responsibility. Indeed, one of the chief concerns about the rise of autonomous machines is that they will enable people to shirk their legal and moral responsibilities to others, in the forward-looking sense, and thereby avoid the duty to compensate or repair harms done, in the backward-looking sense (on these concerns see, e.g. Bryson 2018; Bryson et al. 2017). The rise of autonomous machines thus risks eroding the virtue of taking responsibility and the consequent value of holding people responsible.

There is, however, an alternative perspective to take on all this. In the remainder of this article, I will take that perspective and argue that responsibility is not always a good thing and that techno-responsibility gaps should not always be plugged or dissolved. I will make this argument, in particular, in relation to the alleged virtue of forward-looking responsibility, though it will also apply to the backward-looking form (since the two are closely related). The argument is premised on three claims:

**Claim 1**: Moral choices are often tragic in the sense that they involve irresolvable conflicts between different moral considerations and any attempted resolution of such conflicts necessarily leaves a moral ‘taint’ or remainder.

**Claim 2**: There are different strategies we can use to cope with the problem of tragic choices — *illusionism*; *delegation* and *responsibilisation* — and each of these solutions has its own benefits and costs. None of them is ideal.

**Claim 3**: The use of autonomous decision-making systems presents an opportunity to change the balance of costs and benefits associated with these different strategies. Specifically, it enables a reduced-cost form of delegation, which may give us some justification for moving away from illusionism and responsibilisation and welcoming machine-induced responsibility gaps.

I will defend each these three claims over the next three sections of the article. Although claim 3 is the core of the argument, claims 1 and 2 are necessary precursors to claim 3. If tragic choices do not exist, and if the strategies for dealing with them do not have the mix of benefits and costs that I outline, there is insufficient motivation for claim 3. Spending some time defending those first two claims is, consequently, part and parcel of making the case for claim 3.

## The Problem of Tragic Choice

The first claim is that there are such things as tragic choices. In fact, not only are there such things as tragic choices, they are endemic to human life. We confront them over and over and have to come up with some strategy for coping with them.

For present purposes, I define a tragic choice as a moral conflict in which two or more moral obligations or values compete with one another in such a way that they cannot be resolved or reconciled through decision-making. One of the obligations or values must be traded off against, or sacrificed in favour of, the other. This leads to a moral ‘taint’ or stain on our decision-making and makes moral decision-making a fraught and difficult business. In formulating this definition of a tragic choice, I borrow from the work of Lisa Tessman (2015 and 2017) and Bernard Williams (1973). I want to briefly summarise some of Tessman and Williams’s key ideas here since they form the backbone of my argument in favour of machine-induced responsibility gaps. With one small modification (discussed at the end of this section), I depend largely on their defence of the existence and importance of such tragic choices.

Tessman’s work distinguishes between two kinds of moral conflict: general moral conflicts and moral dilemmas. A moral conflict is any moral decision in which two or more moral obligations or values appear to be in tension with each other. In the case of a general conflict, it is possible that one of the obligations overrides and cancels out the other. Moral dilemmas are a sub-type of conflict. They are decisions in which two or more moral obligations appear to be in tension with each other in such a way that it is not possible to satisfy both obligations at the same time, and neither obligation appears to outweigh or override the other. Medical triage decisions — such as the decisions on ventilator allocation that confronted many physicians in the early days of the COVID-19 pandemic — are perhaps the paradigm case of a moral dilemma. Trolley-type cases, which are frequently used in discussions about the design and operation of autonomous vehicles, are another. These technological variations on trolley-type cases involve an unavoidable vehicle crash where a decision-maker has to pick between programming a vehicle to kill its passengers or to collide with different groups of pedestrians or innocent third parties (Awad et al. 2018**)**.

Some people argue that moral dilemmas do not exist. Such people typically fall into two different groups (Tessman 2017, ch 2). The first group argues that basic principles of moral logic imply that moral conflicts cannot exist and so dilemmas cannot exist; the second group argue that conflicts exist but none of them are genuine dilemmas. The argument made by the first group can be summarised like this: take any alleged dilemma. Imagine a physician with two patients: an 80-year-old and a 50-year-old. She can save one of their lives but not both due to scarcity of time and resources. Ordinarily, we would say that a physician has a duty to save both their patients’ lives. In most cases, it may be possible for them to do so because they have enough time and enough resources. In the triage case, this is no longer true; they cannot save both patients’ lives. That is the heart of the moral dilemma. But you could argue that this dilemma is apparent and not real because there is a general principle of deontic logic that stipulates that ‘ought implies can’. In other words, a person is only obligated to do that which it is possible for them to do; they cannot be obliged to do the impossible. In the triage case, it is impossible for the physician to save both patients’ lives. Therefore, they cannot be obliged to do so. The dilemma cannot exist.

The second group argue against dilemmas by appealing to moral consequentialism. They argue that moral conflicts exist but that in most cases, the tension between two or more moral obligations can be resolved, decisively, in favour of one obligation. For example, although prima facie, it might seem like the physician has an obligation to save both lives in the triage case, a closer inspection reveals that the obligation to save one life outweighs the obligation to save the other. How so? There are different ways to work this out but one is to apply a simple cost–benefit analysis — based perhaps on the number of QALYs (quality adjusted life years), the decision is likely to save. If by saving one life you save more QALYs than another, you have an obligation to save that life. This is a simplistic example, and the rules used to resolve medical triage case are more contested in practice, but the basic point is clear enough; conflicts can be resolved if we examine the facts and likely consequences of our actions in a bit more detail.

These two arguments seem to undermine the idea that tragic choices are real and are endemic to human life. They suggest that either (a) no moral decision-making is tragic because moral conflicts cannot (logically speaking) exist or (b) moral conflict is more apparent than real; moral obligations are negotiable and can be overridden in the right circumstances.

But as both Tessman and Williams argue, this apparent dismissal of tragic choice rings hollow when we consider our own moral choices and their phenomenological impact. Many times, when we are forced to make choices, the moral conflicts feel real and unresolvable. They do not go away even if we rationalise them to ourselves using deontic logic or moral consequentialism. For example, if I give my money to a de-worming charity in the developing world, I cannot give it to a local homelessness charity. I have to pick and choose between good causes. I might use a consequentialist argument to convince myself that the de-worming charity is the better cause — it saves more QALYs per dollar than the homelessness charity — but this does not eliminate the fate of all those homeless people from my mind. They are still real and their suffering is prolonged as a result of my choice.

Williams (1973, ch 11) explains what is going on in these cases by comparing moral conflicts with two other kinds of conflict: conflicts of belief and conflicts of desire. Imagine, first, a conflict of belief. Suppose I believe that I got married on a Friday and I also know that I got married on the 18th of November 2017 (the marriage cert confirms this). Suppose that the 18th of November 2017 was not a Friday, it was a Saturday. Clearly, my beliefs conflict. But this conflict can be easily resolved. I can check to see whether it was a Friday or a Saturday. When I look it up and learn that the 18th of November was, in fact, a Saturday, it will cancel out my earlier belief that I got married on a Friday. Nothing of that belief can remain after the conflict is resolved. Contrast that with a conflict of desire. Suppose I would really like to go to Aruba on my summer vacation but I would also really like to spend my summer on a visiting fellowship at New York University. I cannot fulfil both desires at the same time. In the end, I choose to go to Aruba. Does this mean that my desire for New York dissipates or ebbs away? Not at all. It lingers as a regret. Even if I enjoy my vacation, I will always be inclined to ask: What if I had chosen the other path? The point is that conflicting desires leave ‘remainders’ no matter how you attempt to resolve the conflict. Williams argues that moral conflicts are like conflicts of desire not conflicts of belief. When confronted by a triage case a physician that decides to save one life over another will not simply eliminate from her mind the sense that they violated an obligation to the other person. That sense of obligation lingers. The choice will be a perpetual source of moral regret. It will leave a moral remainder.

This is a largely intuitive argument in favour of the reality of tragic, appealing to our reactions to particular cases. Is there any deeper theoretical reason for thinking that conflicts must leave moral remainders and, more importantly, that they are endemic to human life? Perhaps. Tessman argues that two features of our moral lives lend themselves endemic moral conflict. First, there is the apparent fact of value pluralism. In other words, human values are not, obviously and uncontroversially, reducible to a single scale or type of value (such as, say, subjective pleasure). Instead, values appear to come in many different, apparently incommensurate forms (Mason 2018). We value freedom and equality; security and privacy; life and well-being; health and happiness; and so on. If these values really are plural and incommensurate, then any moral conflict involving a clash of different moral values will tend leave a moral remainder. Why? Because resolving the conflict in one direction does not eliminate or cancel out the neglected moral value. It is not possible to make those kinds of comparative judgment, at least in a rationally defensible and satisfying way.

The second feature of morality is the uniqueness of persons (Zagzebski 2001). It is a common presupposition of moral thought that each human person is both unique and equally valuable. That is to say, no one person’s life takes precedence over another’s and all human lives are distinctly valuable. This implies that human lives are not fungible or interchangeable. This creates problems for any moral conflict involving competing duties to unique persons. Since every person is unique, you cannot trade your obligation toward them with an obligation toward another person. That obligation lingers, no matter what you do. But, when you think about it, many moral conflicts involve competing duties to different people. We often have to decide whether to fulfil obligations to one individual or group of individuals or another, especially when dealing with scarce resources and scarce time.

In short, then, value pluralism and the uniqueness of persons imply that tragic choices are not simply phenomenological in nature; they are an endemic moral feature of human life. We owe obligations to many, distinctly valuable people — our children, our friends, our colleagues, our partners and our fellow citizens — and we work with competing values all the time. This is particularly true of any public decision-making that involves the allocation of scarce resources to different interests, populations or causes. Those allocative decisions always involve weighing up competing values (e.g. healthcare vs education) and affect different, unique people in different ways. This, in turn, is particularly important to bear in mind when it comes to the design of autonomous machine-based decision-making systems. There is a lot of energy behind the use of such machines in assisting important allocative decisions, e.g. in healthcare management and government services (Zerilli et al. 2021). These decisions often bear all the hallmarks of tragic choices. If they are the kinds of decisions associated with potential techno-responsibility gaps, then the argument being defended here should be particularly pertinent to the debate about such responsibility gaps.

This may not be persuasive to everyone. Some may continue to resist the idea that tragic choices are endemic to human life by arguing that both features of morality alluded to above are controversial. For instance, they may argue that value pluralism is false or argue that even if it is true, it does not make it impossible to compare and contrast different moral values. Likewise, some people might dispute the idea that each person is uniquely and equally valuable, as is arguably implied by the deployment of the QALY measure.

It is not possible to resolve these larger philosophical disputes in this article. That said, it may not be necessary to resolve them. Whether value pluralism is true and whether people are, in fact, uniquely and equally valuable may not matter to whether tragic choices pose a significant problem in human life. It may be enough if it *seems* to people that these things are true or that there is uncertainty as to their truth.^2^ The seeming or uncertainty creates tension in moral choices that has a psychological impact and this, in turn, has the power to leave moral remainders. In other words, if it does not seem obvious to you that the value of freedom outweighs the value of well-being, or if you are uncertain whether to rank the former ahead of the latter, you face a decision-making dilemma. If you choose to resolve the uncertainty in some manner, for instance, by using some specific ranking, or favouring a meta-moral decision rule such as maximising expected choice-worthiness (MacAskill and Ord 2020) to favour well-being over freedom, you will not eliminate the moral tension from your mind. You will still be left wondering, given the uncertainty, whether you got the value trade-off right, or whether the meta-moral rule was correct (Nickel 2020). It is likely that only strong moral ideologues, who are convinced that they have a complete and consistent view of moral priorities, can eliminate all apparent tragic choices from their lives. Since few reasonable people are moral ideologues in this sense, and it is not clear that we have good grounds for being ideologues in this sense, tragic choices are likely to be endemic to most humans in most decision-making contexts.

## Three Strategies for Dealing with the Problem of Tragic Choice

If tragic choices are endemic, they pose a significant moral and psychological problem. Morally speaking, they suggest that morally pure decision-making is elusive, that morality is aspirational, and that moral compromise is often essential. Psychologically speaking, they can lead to persistent feelings of guilt, shame, regret and so on. It can be difficult to cope with the psychological and moral burden of tragic choices. We seem to adopt three basic strategies/solutions to the problem of tragic choice: *illusionism*, *delegation* and *responsibilisation*. None of them is a perfect solution to the problem; each has its own costs and benefits. I will discuss them in turn.

### Illusionism

Illusionism^3^is where we convince ourselves that our choices are not tragic or that our decisions leave no moral remainders. In a sense, the arguments outlined in the previous section against the existence of genuine moral dilemmas are examples of the illusionist strategy at work. They are, however, philosophically sophisticated examples of it. There are less sophisticated forms, often aided and abetted by well-known psychological biases. Sometimes we are ignorant of the moral tragedy inherent in our choices, sometimes we are incentivised to ignore the tragedy and sometimes we rationalise away the problem. Psychologists have long-noted that humans have selective attention and engage in motivated reasoning (Driver 2001; Epley and Gilovich 2016; Stanovich 2021). We look for evidence that confirms our preexisting biases and preferences and tend to overlook evidence that does not. This lends itself to illusionist thinking about tragic choices.

This tendency toward motivated reasoning might explain, in part, why the problem of tragic choice is overlooked in some circles. My experience suggests that people can come to accept the problem once they are walked through various examples of it, but few of them see it as a problem that affects their daily lives. Why is this? One reason is that they compartmentalise different aspects of what they do so that they do not have to live with the psychological burden associated with certain moral choices. They claim that they care about climate change, while they take international flights and drive gas-guzzling vehicles. They claim that they care about the moral plight of animals while they feast upon the flesh of factory-farmed pigs. If the moral conflicts inherent in their choices are revealed to them, they engage in other illusionist tricks to eliminate the tension. Through the phenomenon of cognitive dissonance reduction, they work hard to reconcile the competing values or principles inherent what they do, oftentimes by simply pretending that the conflict does not exist that the values are not really in tensions with one another or that they are being perfectly consistent and justified in what they do (Cooper 2019; McGrath 2017). If they are blamed and shamed, they will sometimes pass the blame on to someone or something else. Psychologists have found that when it comes to ascriptions of responsibility, people often commit a ‘fundamental attribution error’: they assume that others bear more responsibility and have more control than they do (Jones and Nisbett 1971; Malle 2006 and 2011). They are quick to blame others and quick to excuse themselves.

Described in these terms, illusionism may not look like a desirable solution to the problem of tragic choice. It is, after all, a form of denialism and ignorance. A moral vice, rather than a virtue. To succeed, it has to suppress the truth about our moral choices. Suppression of the truth can have negative individual and social consequences. William Clifford’s famous essay on the ethics of belief can be read as a critique of non-moral illusionism. Clifford argues that it is ‘wrong always, everywhere, and for anyone, to believe anything upon insufficient evidence’ (Clifford 1877). The type of illusionism I am describing here could be susceptible to a similar critique. We might say that it corrodes someone’s moral virtue if they deny the moral conflict inherent in their choices. In addition to this virtue-based critique, illusionism is also, potentially, a fragile and consequentially harmful solution to the problem. We convince ourselves we are doing the right thing, when in fact we are not. And if a moral conflict is revealed to us over and over again, our psychological defence mechanisms may eventually crumble. This could lead to distress, a sense of hypocrisy and failure, and more.

Despite these costs, illusionism has its upsides. As long as the illusion is sustained, we can avoid the distress and psychological cost associated with tragic choices. We can feel good about ourselves and maintain a sense of optimism and virtue. We can also avoid decisional paralysis. If we persistently ruminated over our moral regrets, we might never get anything done. We might second-guess ourselves too often and avoid difficult decision-making. The moral costs of paralysis could well be worse that the costs of illusionism.

### Delegation

Delegation^4^ is where we get someone (or some *thing*) to make tragic choices on our behalf. We shift the burden of resolving the conflict away from ourselves. Why do we do this? Sometimes the moral conflict is all too real and cannot be imagined away. We mull things over and get stuck in the decision problem. In these cases, passing the buck to another agent can be helpful. They can provide a new perspective on the problem or provide an expertise or disinterest that we lack. Thus, you might wish to delegate medical decision-making to your doctor or legal decision-making to your lawyer. You get them to weigh the factors, assess the evidence, and make a call. This is a form of *reasoned delegation*, where the entity to which you delegate the decision-making engages in a rational decision-making process that replaces (or at least significantly supplements) your own. This, however, is not the only form that delegation can take. We can also delegate to purely arational or random decision-making assistants. For instance, if you are confronted by a really thorny ethical dilemma — e.g. a triage decision where you can only save one of two people that appear to be equal in all respects — you might just flip a coin or roll a dice to help you resolve the ethical conflict.

Couched in these terms delegation has two obvious benefits to individuals: (i) it shifts the psychological and moral costs of the tragic choice away from them and (ii) it shifts them onto an entity that is better able to bear those costs. In doing this, delegation also provides social benefits. To this point, I have been speaking as if tragic choices are, first and foremost, a feature of individual moral decision-making, but, of course, they are not. They are endemic to social, political and legal decision-making too. Indeed, moral conflicts are often most acute and controversial when they arise between people (or groups of people) with competing views. Each individual might be able to sustain their own illusions as to which is the morally correct policy and practice but taken together their views might conflict. We need to resolve that conflict in order to avoid turning it into something more serious. Delegation to a third party is often the most sensible way of doing this. According to Raz’s (1985) theory of legal authority, taking the burden of such decisions away from the individuals involved in the conflict is the key social function of all legal institutions. In doing so, they provide a service, to society, that mediates between individuals and their moral reasons and thereby resolves moral conflicts on their behalf. When they do this successfully, they allow people to bypass moral decision-making altogether.^5^ The authority issues a directive and people follow it.

Consider, for instance, two farmers trapped in a dispute about ownership over a common field. They might agree that the field should be divided fairly and equitably between themselves, but they might have very different conceptions of what fairness and equity entail. Rather than both parties trying to force their moral preferences (if they have clear ones) on the other, the easiest thing for them to do might be to refer the dispute to a legal authority (e.g. an arbitrator). The arbitrator will do the moral reasoning on their behalf and come up with a way to divide the field. They can then substitute the arbitrator’s verdict for their own reasoning, thereby avoiding the moral choice themselves. Indeed, in defending this ‘service conception’ of legal authorities, Raz goes so far as to suggest that such authorities would fail if they did not mediate between individuals and their moral reasons for action. If an arbitrator just told the farmers to divide the land fairly and equitably between themselves, the arbitrator would not be providing an effective service; they would be shifting the moral burden back onto them.

In addition to these individual and social benefits, there is ample evidence to suggest that people are psychologically attracted to delegation when it comes to difficult moral choices. Thinking takes a lot of time and energy. We are predisposed to cut corners whenever we can. Delegation allows us to do this. There are many studies from industrial psychology and human–machine interactions that suggest that people tend towards ‘automation bias’, i.e. deference to the ‘choices’ and recommendations of machines in order to save themselves the cognitive effort (Goddard et al. 2012; Lyell and Coiera 2016; Mosier et al. 1998). These findings support the idea that delegation is psychologically attractive.

Despite its obvious benefits, delegation also has it fair share of costs. We have to be careful to whom we delegate the burden of tragic choice. Reallocating that burden does not eliminate it. In fact, it may just concentrate the burden on particular individuals who suffer more greatly as a result. An apt illustration of this, in the present context, might be the psychological plight of ‘content moderators’ on social media platforms such as Facebook. As is well-known, Facebook employs thousands of human beings to review content shared on its platform to ensure that it meets certain standards (no child pornography, etc.). They effectively serve as arbitrators of speech on the platform. According to reports, being a content moderator is a stressful and difficult job (Au-Yeung 2021; Criddle 2021; Newton 2019). They are under a lot of pressure and are required to review extremely offensive and often traumatising content. They save us from having to do so, but at a cost to themselves. In addition to this, if we always delegate to others, we never develop the virtue of moral agency in ourselves. If we delegate too readily, our moral ‘muscles’ may atrophy. We may never do the hard work of trying to figure out what is right or wrong for ourselves. This could lead to a slippery slope in which we extend the delegation strategy to all our choices, not just the tragic ones (Vallor 2014).

###  Responsibilisation

Responsibilisation is where we confront the tragic choice head-on and accept responsibility for resolving the conflict in a particular way. We do not pass the buck to someone else; we do not bury our heads in the sand. We embrace the fact that life sometimes involves these tragic tradeoffs and we live with the consequences. We accept that it is our moral responsibility to decide.

Expressed in these terms, responsibilisation might strike many people as the preferred solution to the problem of tragic choice. As noted earlier, we tend to see taking responsibility, and the moral agency that enables us to take responsibility, as virtues (Ruyter 2002; Williams 2008; Torrance 2021). There are long-standing traditions in philosophy that tell us that it is good to cultivate sensitivity to moral reasons, to exercise practical reason and to take responsibility for one’s choices. Furthermore, and as noted earlier, firm belief in the goodness of responsibility is implied in most discussions of techno-responsibility gaps. The motivation behind those discussions is that if machines open up a responsibility gap, then we lose something of value.

People also think, readily, in terms of responsibilisation. According to several theories of the psychological and cultural origins of morality, practices of responsibility and punishment have played a central role in the emergence of the human moral conscience (Boehm 2012; Tomasello 2016; Wrangham 2019). For instance, Michael Tomasello has argued that human moral psychology first started to emerge when humans engaged in complex, coordinated behaviours (such as big-game hunting). When we did that, we started to develop a conception of ourselves as occupying a certain social role with associated duties and responsibilities. If we or another person failed to live up to those responsibilities, we subjected ourselves or them to moral critique and punishment. In time, this practice generated a rich underlying moral psychology consisting of reactive attitudes (anger, resentment, shame, etc.) and beliefs (X has failed to do his fair share; Y is not trustworthy, etc.). Admittedly, and as noted earlier, we tend to be quicker to blame others than we are to blame ourselves, but it is possible to turn this tendency towards blame around on ourselves too and see ourselves as proper subjects of blame and punishment.

For all its merits, however, responsibilisation has a dark side. In our zeal to find one another responsible, we can blame too quickly, ignore mitigating factors, impose harsh punishment and draw unjustified conclusions about the capacity of people to control their choices. Free will skeptics routinely point out these problems, highlighting the fact that none of us is in complete control of our lives and that we are often made to suffer unjustly, both psychologically and physically, through practices of blame and punishment (Levy 2011 and 2014; Pereboom 2001 and 2014; Waller 2011, 2015 and 2017**)**. Some such skeptics go so far as to argue that de-moralising our failures, and opting for a medicalised harm-reduction approach to wrongdoing would be more socially beneficial (Caruso 2021). One does not have to accept all of their criticisms to see that they have a point.

Fears about the dark side of responsibilisation are particularly important to bear in mind when dealing with tragic choices and techno-responsibility gaps. If it is true that moral conflicts cannot be resolved, and that they inevitably leave moral remainders, there is a danger that we blame people for resolving the conflict in a manner in which we disapprove. In other words, appealing to responsibilisation, particularly in the context of tragic choices, is all too often an invitation to ignore the tragedy involved in those choices and to punish people unfairly for not following our moral intuitions. Likewise, novel technologies such as autonomous machines often create uncertainty as to who is the proper bearer of responsibility which can, in turn, lead to illegitimate scapegoating and punishment. For instance, Elish (2019) has argued that autonomous driving systems can create ‘moral crumple zones’ in which responsibility for harmful outcomes is unfairly attributed to the human users of these systems, who typically have no say in their design and operation, and not to the owners and controllers of the technology. A rush to responsibilisation, in response to a perceived techno-responsibility gap, could consequently do more harm than good.

### Summary

To reiterate, none of these solutions is ideal. Each has its benefits and costs. Illusionism seems like the least attractive solution from a moral perspective since it appears to reward ignorance. That said, it is a psychologically attractive and common coping mechanism. Delegation and responsibilisation both have moral benefits, but also come with significant potential costs. Given the nature of tragic choice, we will never find a perfect solution to the problem. We simply need to select the right balance of strategies. Sometimes, this may lead us to favour delegation over responsibilisation; sometimes, it may lead us in the opposite direction.

**Table:** 

Solution	Benefits	Costs
Illusionism	Reduces psychological stress, helps to maintain sense of optimism and moral rectitude	Rewards ignorance and avoidance of truth; unstable and fragile
Delegation	Shifts psychological and moral burden onto an entity that is better able to bear that burden; reduces psychological stress	Corrosion or atrophy of moral agency; excessive concentration of stress and moral burden on particular individuals and institutions
Responsibilisation	Develops moral agency and virtue; accepts reality of tragedy and embraces compromise	Danger of scapegoating; assuming more responsibility than we rightfully deserve to bear; psychological and physical distress of excess blame and punishment

## Why We Should Embrace (Some) Techno-Responsibility Gaps

So far, I have argued that tragic choices are endemic to human decision-making and that we have three main strategies for coping with this tragedy. Each of these strategies has both benefits and costs. With those claims in place, I can, at last, turn to the main part of my argument that one potential advantage of machines is that they allow for a positive shift in the balance of strategies we use to address the problem of tragic choice, but they only do so if we embrace, rather than lament, the fact that they open up responsibility gaps. To put it another way, there are times when, confronted with a tragic choice, we should favour delegation to autonomous systems without, necessarily, striving to fill the responsibility gaps that may be opened up as a result of that delegation.

The core argument for this view can be easily stated. As noted above, delegation is sometimes a beneficial way in which to solve problems of moral conflict. It saves individuals from bearing the moral and psychological cost of dealing with ineliminable moral conflicts and it is a useful and, indeed, expected way of addressing moral conflict at a societal level. But when we delegate to other humans or human-run institutions, this comes with moral and psychological costs. We can expect too much of them, and concentrate excess psychological distress in their hands (think, again, of the plight of the content moderators). The advantage of delegation to machines is that it gives us the benefits of ordinary delegation without the typical associated costs. Machines do not have to suffer the psychological and moral distress of human agents. They can resolve conflicts without bearing the burden. However, this reduced-cost form of delegation is only possible if we accept that it will result in a type of responsibility gap; the machine cannot be responsible, nor is it appropriate or desirable to trace responsibility to us.^6^

This framing may suggest that the value of delegation lies, primarily, in removing the costs associated with backward-looking responsibility, but this is not the case. Much of the value lies in removing the expectation of forward-looking responsibility from human decision-makers. In other words, the cost reduction arises when we say to ourselves and others that it is not our responsibility to make this choice nor to bear the moral costs typically associated with it. This has consequences for backward-looking responsibility. It makes seeking backward-looking responsibility illegitimate and hence removes the costs associated with it, but this is only possible, only if we first accept the value of the forward-looking responsibility gap. It is the absence of perceived obligation or virtue in taking responsibility for tragic choices that discourages the tendency to do so. This, in turn, legitimises the attempt to eliminate the moral and psychological costs associated with delegation.

Obviously, this argument comes with a number of caveats. First, the argument is not claiming that delegation to machines is always a good thing. The argument focuses on delegation in the case of tragic choices only. Not all decisions meet the threshold for a tragic choice. Still, as already noted, many allocative social decisions, in which we have scarce resources and competing interests, do involve tragic choices and hence the argument is of broad social applicability.

Second, although the argument is of broad applicability, it does not follow that we should delegate decision-making authority to machines recklessly or without due consideration of the consequences of doing so. We should only delegate when we are confident in the reliability of the system and we are confident that it engages with the moral tradeoffs inherent in the choice.

Third, although the argument claims that machine delegation is reduced-cost, it does not claim that it is cost-free. The argument focuses on the costs associated with bearing the burden of choice. There are other associated costs. As noted, one concern about delegation is that it might corrode or atrophy our own moral agency. If we are always delegating to machines, we never give ourselves the space to develop and maintain our own moral agency. If we think moral agency is a virtue — and nothing in this article denies that view — we have to make sure we do not overdo delegation to machines. It is about finding the right opportunities for delegation and the right balance of delegation vs responsibilisation. Determining the right balance will depend on factors that are specific to societies and individuals. What are the tragic choices we find most difficult to resolve and in which machine assistance could be beneficial? Do we still give ourselves plenty of opportunities to develop our moral agency? These questions will have to be answered on a case-by-case basis. The only point being made in this article is that autonomous machines create a moral opportunity for reduced cost delegation that can be overlooked if we constantly seek to fill responsibility gaps.

Fourth, the argument assumes that autonomous machines are not capable of suffering the psychological distress associated with tragic choices. This seems like a safe assumption, at least given present-day technology. There is nothing to suggest that present day robots or AI systems suffer some inner psychological turmoil. Nevertheless, it may be possible that some machines do, someday, experience such distress and thus bear the same moral and psychological burden as a human to whom a tragic choice has been delegated. The argument will not apply to those machines. We may still wish to delegate to such machines, but it will not come at a reduced cost.

In addition to these caveats, there are also several more substantive criticisms of the argument. I will close by considering four of them.

First, one might argue that we do not need sophisticated autonomous machines to avail of a reduced-cost form of delegation. Perry and Zarsky (2015), in a fascinating exploration of the role of randomisation and lotteries in legal decision-making, argue that in certain scenarios, when moral interests and considerations seem equally weighty on both sides of a dispute, and there are pressures of time and scarce resources, the use of randomisation has been, and often should be, embraced by social institutions. Disaster cases are a striking and popular example of this. Imagine three sailors stranded on a life-rafting that is sinking due to their combined weight. If one of them is thrown overboard, the other two have a chance to survive. If not, they all die. How should they decide who should live and who should die? Drawing lots is a quick and palatable solution to the moral conflict. It involves no moral evaluation of the respective worth of different lives. It is not biased in any way. It is purely random. Given that my argument about machine-based delegation appeals to conflicts of this sort, we might be tempted to suppose that we do not need to delegate to machines in tragic cases. We can just use some lottery or random allocation system.

This is an interesting criticism. I agree that there are many instances when we should embrace randomisation in public decision-making. But one can embrace this whilst still supporting the argument outlined above. For starters, machines can help with randomisation, particularly if we are trying to randomise over many possible outcomes or people. Pseudo-random number generation and automated lotteries are an efficient way of handling large-scale randomisation. It is often difficult for humans to randomise and manage large numbers of possible outcomes. So there are cases in which randomisation and machine-delegation go hand-in-hand. Still, there are other cases in which delegation is desirable but we do not want a purely random decision-making system. We want reasoned delegation; some attempt to weigh the competing moral values and obligations. This is likely to be true in cases in which the moral values or obligations are not as finely balanced as they are in the triage or shipwreck cases discussed above, or where the set of relevant moral considerations is vast and inaccessible to the human mind. Consider, for instance, a case in which we need to decide how to allocate funding to a set of different hospital systems or medical technologies. There are many variables to consider, every choice will come at some moral cost, but they are not all obviously equal or equivalent. In those cases, machine delegation could be particularly useful. One of the virtues of machine learning systems, for instance, is that they can integrate vast pools of data and spot patterns and possibilities in that data that humans cannot (Robbins 2019).

Second, one might argue that it is impossible to reduce the costs of delegation in the way that I suppose. If we do delegate a tragic choice to a machine, we still must choose whether to allow that delegation or not (i.e. we must take and bear responsibility for the decision to delegate). So even if we are not directly causally responsible for what the machine does, we are responsible for the decision to delegate. We might experience some sense of distress or regret for doing this, particularly if the machine’s output is unwelcome or harmful to some people, and we may, consequently, be blamed for this decision to delegate. In a sense, this is what many discussants in the ‘techno-responsibility gap’ literature propose as a resolution to the problem of techno-responsibility gaps. They favour tracing responsibility for autonomous machines back to the humans that decided to design and deploy those systems. Their argument is that responsibilisation remains viable, perhaps in a slightly different form, even after we delegate to machines.

A novel variation on this argument, which reaches an even stronger conclusion, has recently been put forward by Di Nucci (2021, ch 12). He claims that it is impossible to delegate responsibility to machines. Di Nucci’s argument is complex. He does not argue that delegation changes nothing about responsibility and he accepts that delegation can sometimes result in different agents sharing responsibility for a given outcome. Nevertheless, he insists that in choosing to delegate, the delegator does not lose any responsibility for the outcomes achieved by the delegatee. His argument is developed through the intuitive analysis of a series of worked examples. Here is a variation on one of them: Imagine you have a hospital management team that needs to make complex ethical choices about the allocation of healthcare resources (e.g. how much to invest in different kinds of treatment/technology for a range of different illnesses).^7^ They can make this decision themselves or they can outsource the decision to a machine. Suppose they choose the machine and, later, something goes wrong. Questions are raised about responsibility for the resulting allocation. Would the management team avoid responsibility simply because the machine decided on the final allocation? Di Nucci’s intuition is that they would not and he supports this by asking us to imagine the counterfactual scenario in which they made the choice themselves. If they made the choice themselves, they would retain responsibility. Why should it be different if they delegated? Sure, there may have been some glitch in the machine, or some design flaw in its software, and that may implicate the manufacturers of the machine, but the management team still made the choice about how best to resolve the allocative dilemma. They chose to resolve it through delegation. They retain responsibility for the allocation that followed from that choice.

There is some validity to this criticism. Every choice we make is an opportunity for responsibilisation. It is possible to take responsibility and hold someone (or some group) responsible for the decision to delegate a tragic choice to a machine. But the stronger claim that there is no net loss of responsibility, or, more importantly, that there *ought* to be no net loss in responsibility strikes me as implausible. There are three reasons for this. The first reason is where there is delegation; there is some causal and moral distance between the decision to delegate and the tragic choice. A decision to delegate can be fully justified and blameless in a way that a decision to resolve a tragic choice may not be; and vice versa. Consider, once again, Raz’s (1985) service conception of legal authority, the decision to delegate to the arbitrator in cases of moral conflict is, in many instances, the most morally desirable and efficient choice, but it is sealed off from the arbitrator’s resolution of the conflict. That sealing off is the whole point of using the arbitrator. The second reason is that sensitivity to the tragedy of such choices should disincline us towards an excess of blame and shame over the decision to delegate. Indeed, fostering such a disinclination is one of the motivations behind the argument being defended in this paper; sometimes there is good reason to support delegation, at least in the case of tragic choices. The third reason, which is related to the second, is that in the case of a tragic choice there arguably should be no responsibility for the outcome in the absence of delegation, particularly if the choice involves a complex dilemma. This point is particularly relevant to Di Nucci’s argument. Di Nucci’s claim that there is no loss of responsibility through delegation is plausible if we assume that there ought to be responsibility for tragic choices in the first place. But part of the argument I am making is that this should not be the case. Tragic choices are hard, if not impossible, to resolve and it is often unfair and unjust to expect someone to take responsibility for them or to punish and blame them for the outcome. The problem with our current social practices of responsibility is that we tend to have those expectations and they filter into the phenomenology of tragic decision-making (I feel guilty, no matter what I do) and the social treatment of those that make such decisions. Autonomous machines could be the disruptive force we need to break this link.

Third, one might counter that the argument will provide an excuse for agency-laundering and/or liability evasion. Agency-laundering arises whenever someone obscures their moral and causal responsibility for an outcome by delegating or outsourcing decision-making authority to another entity. The potential for autonomous machines to enable agency-laundering has been articulated by Rubel et al. (2019). They argue that agency-laundering is distinct from, albeit related to, techno-responsibility gaps.^8^ In brief, their claim is that doubts over the responsibility of autonomous machines, coupled with beliefs in their neutrality, complexity and opacity, will enable nefarious actors to ‘launder’ their moral agency through those machines and obscure their causal responsibility for harmful or morally undesirable outcomes. As an example of this, they mention Facebook’s^9^ reluctance to accept responsibility for facilitating anti-semitic ads on its platform through an algorithmic recommendation system. When the problem of anti-semitic ads was revealed to Facebook, they vowed to change their practices, but they denied any retrospective responsibility for what happened. They did this by appealing to the complexity of the algorithmic recommendation system. This, Rubel et al. suggest, is an all-too-convenient excuse given (a) Facebook’s control over the use of such algorithms and (b) the fact that such ads benefitted its bottom line. As they put it:

Facebook’s business model includes allowing advertisers to target groups of people narrowly and effectively. It does this in a way that avoids the labor costs associated with human approval of ad targets or human oversight of ad categories and purchases. In so doing, Facebook implies that its algorithmically-generated categories and suggestions are relevant to advertisers (otherwise, advertisers would have no reason to purchase ads).

(Rubel et al. 2019, 1026).

Since the argument defended in this article claims that it is sometimes desirable to disavow responsibility for complex moral choices, and foist them onto complex and opaque machines instead, one might worry that it provides a smokescreen for similar exercises in agency-laundering.

There are several things to be said in response to this. First, whether agency-laundering is, in fact, a problem depends on whether we think people ought to take responsibility and/or be held responsible for certain choices. As outlined in response to the previous objection, this is arguably not the case when it comes to tragic choices. These choices are hard, they have no obvious morally correct answer, and the psychological and social costs of responsibilising those choices can be significant. If delegation can reduce those costs, then there is no moral problem of agency-laundering. There is nothing of equivalent value being laundered through the machines. Second, notwithstanding this point, there is undoubtedly a potential for nefarious actors to dodge responsibility by claiming that they were dealing with a tragic choice and hence they were justified in delegating decision-making authority to a machine. We should do our best to prevent this from happening. The decision to delegate a tragic choice to a machine should not be taken lightly. It should be subject to appropriate deliberation and discussion. Does it really involve a tragic choice? Is it a case where the loss of agency is outweighed by the benefits of delegation? These questions should be asked and answered in a transparent manner prior to any decision to delegate. Third, if the decision to delegate results in some significant and unanticipated social harm, this could be addressed through the use of social insurance funds that compensate the affected without applying a liability burden to an individual. Indeed, one could argue that the greater use of social insurance funds, as opposed to traditional legal tools of responsibility and liability, is an important implication of the argument being defended in this article.

Fourth, and finally, one might object that delegation to machines does not reduce the burden of tragic choice, it actually heightens it by making tragic trade-offs more apparent and less easy to ignore. An argument to this effect has emerged from recent debates about fairness in algorithmic decision-making. The motivating example is well-worn so forgive me for repeating it here. In 2016, concerns were raised about the use of algorithmic risk scores in the US criminal justice system. A number of US states used a proprietary system (known as the COMPAS algorithm) to score the recidivism risk of defendants and prisoners in order to decide whether to release them on bail or release them on parole. A study by ProPublica journalists suggested that these algorithms were systematically biased against black defendants/prisoners, consistently resulting in more false positive risk scores for them than for white defendants/prisoners (Angwin et al. 2016). This appeared to be clearly unfair.

The ProPublica study, however, led to a number of responses from the creators of the algorithm and subsequent debates and analysis by teams of academics. Without getting too immersed in the details, one of the key insights that emerged from the debate is that there are a number of different criteria/values that we might like a fair decision-making system to fulfil (Binns 2018). It turns out that it is not possible to satisfy each of those criteria at the same time. In other words, our conceptions of fairness are multidimensional and it is impossible to optimise decisions across each dimension of fairness (Kleinberg et al. 2017; Heidari et al. 2019). In some ways, this is a good formal illustration of the prevalence of tragic choices.

Interestingly, though, defenders of algorithmic decision-making argued that one potential virtue of such systems is that they make such tragic choices more salient, encourage responsibilisation and disincline us from illusionism (Kleinberg et al. 2018**)**. Their insight was that, right now, when we rely on human decision-makers to determine what is fair, we often implicitly accept that there will be some fudging and compromise involved. This fudging takes place inside the minds of the human decision-makers and largely goes unquestioned. If we use algorithmic risk predictions, we have to make this compromise explicit and precise. We can no longer pretend that the decision has successfully balanced all the competing interests and demands. The reason for this is that we have to be explicit about the tradeoffs when we translate the moral choice to some formal, machine-readable code. This could be seen as a benefit. A major problem at the moment is that much human decision-making is discriminatory in the sense that it makes tradeoffs across different dimensions of fairness, but its discriminatory nature is implicit and hidden from view. The widespread use of algorithms forces those discriminatory tradeoffs into the open where they can be washed by the great disinfectant of sunlight. We can no longer hide behind the fuzziness of human decision-makers. In choosing to delegate to a machine, we have to accept responsibility for the tradeoffs inherent in our choices. So, far from reducing the costs of tragic choice, machine delegation may increase it.

I happily concede some ground to this criticism. I agree that it is possible to design autonomous decision-making systems in such a way that they render tragic choices more explicit and salient. One could see this as an advantage because it lifts the veil of illusionism and pushes us toward greater responsibilisation. But this is not the only way in which to design such systems, nor is such transparency or explicitness always a virtue. We can design autonomous systems that integrate multiple dimensions of variance into a single decision without making it clear how they balance or weigh those dimensions. In other words, we can design those systems to be fuzzy or opaque, in much the same way as human decision-makers can be fuzzy or opaque. This fuzziness is, in fact, a feature, not a bug, of some machine learning algorithms; their whole reason for being is that they can be trained to produce a certain kind of decisional output without it being clear to their programmers or users how they weigh all the variables relevant to producing the output (Robbins 2019).

Ironically, of course, the unintelligibility or opacity of machine learning systems is typically thought to be a major problem with their ethical deployment. It has led to demands for machine explainability and, at least in the European context, specific legal rights (as part of the General Data Protection Regulation) that support a kind of ‘right’ to explainability. Full engagement with the explainability literature would take us too far afield but there is some dispute about the exact form or standard of explainability that is appropriate. The only point I wish to make here is that, in the case of tragic choices, making tradeoffs more explicit and transparent is not always a good thing. Sometimes we might favour the design of a fuzzy or opaque system precisely because it enables a reduced-cost form of delegation. Just as responsibility is not always a virtue so too is explainability not always a virtue.

All of this might, however, suggest that there is a meta-tragedy at the heart of the argument put forward in this article. We face many tragic moral choices. We have a range of strategies we can follow when making those choices, each of which has its own costs and benefits. The selection of one strategy in favour of another is thus, in itself, a kind of tragic choice. If we pick delegation over responsibilisation, we do not completely eliminate the allure of responsibilisation. But this meta-tragedy is a feature of my argument, not a bug. The point is that we have to deal with tragic choices but that there are ways to reduce their moral burdens. Delegation to machines, at the expense of human responsibility, might be one way to do this.

## Conclusion

In summary, in this article, I have defended an alternative perspective on techno-responsibility gaps. Although the prevailing wisdom seems to be against such gaps, and the policy proposals tend to try to find ways to plug or dissolve such gaps, I have argued that there may be reasons to welcome them. Tragic choices — moral conflicts that leave ineliminable moral remainders — are endemic in human life and there is no easy way to deal with them. We tend to cycle between different responses: illusionism, delegation and responsibilisation. Each of these responses has its own mix of benefits and costs. None of them is perfect. That said, one potential advantage of advanced autonomous machines is that they enable a form of delegation with reduced moral and psychological costs. Thus, they can shift the balance of strategies in favour of delegation and away from responsibilisation. This is only true, however, if we embrace the resultant techno-responsibility gaps. I am fully aware that this position goes against the grain and is contrary to emerging law and policy on autonomous systems. I offer it as a moderate corrective to the current consensus. Responsibility gaps are not always a bad thing. Delegation to machines, particularly in the case of difficult tragic choices, might sometimes be a good thing.

## Data Availability

Not applicable.

## Footnotes

1 The specific term, ‘techno-responsibility’ gap, is taken from Tigard (2021)

2 The recent debates about moral uncertainty and its practical significance might be pertinent here, see for example MacAskill and Ord 2020 and, relatedly, Nickel 2020 on the moral uncertainty caused by novel technologies. Nickel’s arguments are particularly interesting in the present context because they suggest that novel technologies, if they are sufficiently disruptive of classical moral reasoning, can create even more tragic choices by inducing greater uncertainty about the morally correct course of action.

3 ‘Illusionism’ is also a well-known position in the free will debate. It is defended by Saul Smilansky (2000) and it holds that we pretend that we have free will (we sustain the illusion of free will) even in the face of evidence of causal determinism.

4 Again, similar to ‘illusionism’, ‘delegation’ is not the ideal term. There is large literature on the ethics and legality of delegation in public decision-making. Most of those discussions establish certain conditions that need to be satisfied in order for a morally acceptable form of delegation to take place. Typical among such conditions is an *accountability* condition that insists that the delegator is accountable for the decisions of the delegatee. My use of the term ‘delegation’ is intended to be read without these moral connotations. In other words, for my purposes delegation could take place without residual accountability. For a discussion of delegation in the context of AI, see Zerilli et al (2021), chapter 11 and Coglianese and Levy (2017)

5 In saying that they ‘allow’ for moral reasoning to be bypassed, it does not follow that they necessitate such bypass. People may still wish to engage in their own moral reasoning and there may still be reasons to morally scrutinise the decision to delegate to the legal authority.

6 Cf Tigard 2021 who, as noted earlier, might argue that this means that there is no ‘gap’ that needs to be filled in these cases. In a way, the argument defended here could be interpreted as being compatible with Tigard’s attempt to ‘dissolve’ the techno-responsibility gap problem. That said, my argument is a more explicitly axiological one; it is not just that there is no morally legitimate demand for responsibility in this case; it is also that the absence of such a demand is a good thing. Something to be welcomed and not lamented.

7 This example is my own formulation, not Di Nucci’s. I think it captures the essence of his examples but it is deliberately different. I have explicitly assumed that the allocative decision is a tragic moral choice, involving the weighing and contrasting of complex moral variables, and not a more straightforward moral choice. Most of Di Nucci’s examples involve more straightforward moral choices.

8 It is not entirely clear that they are successful in this since their concern about agency-laundering appeals to concepts that are tightly connected to the techno-responsibility-gap debate. They may succeed in articulating a distinctive version of the techno-responsibility gap concern.

9 The parent company has since been renamed ‘Meta’ but the platform in question was, and still is at the time of writing, called Facebook.
